# White matter tracts for the trafficking of neural progenitor cells characterized by cellular MRI and immunohistology: the role of CXCL12/CXCR4 signaling

**DOI:** 10.1007/s00429-014-0770-4

**Published:** 2014-04-26

**Authors:** Chiao-Chi V. Chen, Yi-Hua Hsu, D. M. Jayaseema, Jeou-Yuan Joanne Chen, Dueng-Yuan Hueng, Chen Chang

**Affiliations:** 1 N123, Institute of Biomedical Sciences, Academia Sinica, 128, Section 2, Academia Road, Nankang, Taipei, 11529 Taiwan; 2 Department of Neurological Surgery, Tri-Service General Hospital, National Defense Medical Center, 325, Section 2, Cheng-Kung Road, Neihu, 11490 Taipei, Taiwan; 3 Department of Biochemistry, National Defense Medical Center, Taipei, Taiwan

**Keywords:** SDF, CXCR4, Stem cell, Migration, Tropism, White matter, Neurogenesis, Homing, Directed migration

## Abstract

White matter tracts are important for the trafficking of neural progenitor cells (NPCs) in both normal and pathological conditions, but the underlying mechanism is not clear. The directionality of white matter is advantageous for molecules or cells to distribute over a long distance, but this feature is unlikely solely responsible for efficient migration. The present study hypothesizes that the efficient migration of NPCs into white matter is under the influences of neurochemical attraction—CXCL12/CXCR4 signaling, a major mechanism underlying the targeted migration of NPCs. To test this view, the present study investigated the effects of CXCL12 administration into the corpus callosum (CC) on the migratory behavior of transplanted NPCs. A living animal tracking platform based on MRI and a magnetic cell labeling technique was employed. The NPCs were magnetically labeled and then transplanted at the right end of the CC. CXCL12 was infused continuously at the left end. Migration of NPCs was monitored repeatedly over a 7-day course using 3D gradient echo T2*-weighted imaging. It was found that, CXCL12 induced NPCs to migrate up to 1,881 μm from the graft whereas the spontaneous migration was mere 200 μm. CXCL12 induced migration that was nine times as efficient in the speed. The results indicate that the CXCL12/CXCR4 signaling may be a mechanism via which NPCs efficiently migrate along the white matter tracts. The study also presents a potential strategy for facilitating the targeted migration in NPC therapy for brain disorders.

**Electronic supplementary material:**

The online version of this article (doi:10.1007/s00429-014-0770-4) contains supplementary material, which is available to authorized users.

## Introduction

White matter tracts, which mainly involve transmitting neural signals from one brain region to another, are important for the trafficking of neural progenitor cells (NPCs) (Suzuki and Goldman 2003; Yang et al. 2009). Both endogenous and transplanted NPCs exhibit the tendency to take advantage of the white matter tracts for migration regardless of the condition being normal or pathological. Magnetic resonance imaging showed that, in neonatal rats, endogenous NPCs in the subventricular zone labeled by micron-sized paramagnetic iron oxide (MPIO) moved into the corpus callosum (CC) to reach the cortical and other regions within 7 days (Yang et al. 2009). Using immunohistological approaches, it was shown that transplanted NPCs migrated extensively in the corpus callosum, internal capsule, and hippocampal fiber tracts in the neonatal rat brain after being deposited into the striatum (Zhang et al. 2001). In the brain of ischemic stroke, transplanted NPCs migrated over the CC to reach the infarct site at an estimated speed of 360 μm/day (Kim et al. 2004).

Little is known regarding the mechanism via which NPCs tend to utilize the white matter tracts as a migration route. Structurally, white matter is highly directional for water diffusion, as indicated by diffusion tensor MRI (Assaf and Pasternak 2008). It is evident that white matter, as compared to the gray matter, is more advantageous to enable molecules or cells to distribute over a long distance. However, the migration speed of engrafted NPCs through the CC was estimated to be 50–70 μm/day in the normal condition (Flexman et al. 2011). Such spontaneous migration is relatively slow, suggesting the directional diffusion is unlikely solely responsible for the faster migration reported in many of the related studies (Kim et al. 2004; Yang et al. 2009; Zhang et al. 2001).

The present study hypothesizes that efficient migration along the white matter structures is under the influences of neurochemical attraction. It is well established that chemotaxis via CXCL12/CXCR4 signaling mediates the targeted migration of NPCs toward brain lesion. Studies have indicated that the increased CXCL12 levels of the injured site form a concentration gradient that attracts cells bearing the receptor to migrate along (Tiveron et al. 2006; Liapi et al. 2008; Robin et al. 2006; Itoh et al. 2009; Imitola et al. 2004; Stumm and Hollt 2007; Belmadani et al. 2005, 2006; Bhattacharyya et al. 2008; Aboody et al. 2000; Banisadr et al. 2011; Takeuchi et al. 2007). NPCs, which extensively express the cognate receptor CXCR4 on the cell membrane, represent a major population being attracted by the CXCL12 elevation (Itoh et al. 2009; Peng et al. 2007). However, unlike the injured site, the white matter tracts are less concerned with regard to CXCL12 release in many of the studied conditions (white matter diseases being the exception). With little information available about CXCL12 expression in the fiber tracts, the role of the CXCL12/CXCR4 axis in the rapid migration through the white matter structure by NPCs remains to be investigated.

To the end, the present study employed a living animal tracking platform based on MRI and a magnetic cell labeling technique. The NPCs were magnetically labeled and then transplanted at the right end of the CC in adult rats. CXCL12 was infused continuously at the left end to form a concentration gradient. Migration was tracked by three-dimensional (3D) gradient echo T2*weighted imaging (T2*WI) repeatedly for up to 7 days. The temporal and spatial characteristics obtained from cellular MRI were used to characterize NPC migration along the CC. Immunohistology was also used to corroborate the MRI findings. The study used adult rats for investigations because minimal spontaneous NPC migration has been reported. The findings of the study present not only an understanding in the use of white matter as a migratory route by NPCs, but also a potential strategy for facilitating the targeted migration in NPC therapy for brain disorders.

## Materials and methods

### Animal subjects

Twenty-three adult male Sprague-Dawley rats purchased from National Laboratory Animal Center of Taiwan were used. Twelve rats were used in the first experiment, which was designed to validate the administration protocol (vehicle-treated, *n* = 6; CXCL12-treated, *n* = 6). Eleven rats were used in the cellular MRI experiment (vehicle-treated, *n* = 5; CXCL12-treated, *n* = 6). The rats were 8–10 weeks old and weighed 250–300 g before experiments. Rats were housed in threes in plastic cages with free access to food and water. The housing environment was specific-pathogen-free with a 12:12-h light: dark cycle and controlled humidity and temperature. All experimental procedures were approved by the Institute of Animal Care and Utilization Committee at Academia Sinica, Taipei, Taiwan.

### Primary NPC culture

Primary NPCs were harvested from D14 embryonic brain tissues of C57BL/6 pregnant mice purchased from National Laboratory Animal Center of Taiwan. The tissues were digested by collagenase type 2 (Sigma-Aldrich, St. Louis, MO, USA) at 2 mg/mL for 2 h at 37 °C. The cells were plated at a density of 1.5 × 10^6^ cells/mL and propagated as free-floating neurospheres in the N2 medium consisting of Dulbecco’s modified Eagle’s medium and Ham’s F-12 medium (DMEM/F-12) and 1 M HEPES (both from Gibco, Invitrogen, Grand Island, NY, USA), 0.285 % d-glucose, 23 μg/mL insulin, 92 μg/mL apo-transferrin, 8.9 μg/mL putrescine, and 75 μM sodium selenite (Sigma-Aldrich), 20 ng/mL human recombinant epidermal growth factor (BD Biosciences, San Jose, CA, USA), and 20 ng/mL FGF-2 (Sigma-Aldrich). Upon reaching of the sphere size to 140–150 μm, the cells were passaged by mechanical trituration with a fire-polished Pasteur pipette (Corning, Tewksbury, MA 01876, USA) through a 40-μm cell strainer (BD Biosciences). The standard cell culture atmosphere was maintained with 5 % CO_2_ at 37 °C throughout the experiments.

### Flow analysis

Aliquots of 1 × 10^6^ viable cells were incubated in PBS with anti-mouse CXCR4 (1:100; eBioscience, San Diego, CA, USA) antibody conjugated with allophycocyanin (APC) and 1 % fetal bovine serum (FBS) (Gibco) for 15 min in dark at 4 °C. After three washes, the cells were resuspended in PBS containing 2.5 μg/mL of 7-aminoactinomycin-D (7-AAD; BD Biosciences) and 1 % FBS for 10 min at room temperature. Flow cytometry was performed on FACSCanto (BD Biosciences). A total of 10,000 counts were acquired for each analysis using FACS diva v 6.1.2 (BD Biosciences). The experiment was repeated three times.

### Magnetic NPC labeling with MPIO

For magnetic labeling, NPCs were suspended in PBS with 150 μg/mL MPIO (0.96 μm; catalog # MC03N; Bangs Laboratories, Inc., Fishers, IN, USA) and then received electroporation of a single pulse at 0.17 V for 200 ms followed by overnight incubation using Gene Pulser (Bio Rad, Richmond, CA, USA). After three washes to remove free MPIO, NPCs were counted and used for experiments. For each labeling, 1 × 10^5^ cells were spared to verify the labeling procedure using Prussian blue (PB) staining as described below. On average, 81.7 ± 2.7 % (mean ± standard deviation) of the cells were positively labeled with MPIO.

### NPC engraftment and CXCL12 infusion

The rats were anesthetized intraperitoneally by chloral hydrate (Sigma-Aldrich) at 450 mg/kg. 1 × 10^6^ MPIO-labeled NPCs in a volume of 5 μL were injected into the right end of the CC (AP 1.0 mm; ML +1.2 mm; DV −3 mm) of the brain on a stereotaxic apparatus (Stoelting, Wood Dale, IL, USA). The injection rate was 1 μL/min by a micro-infusion pump (Model: Pump 11 Elite from Harvard Apparatus, Holliston, MA, USA). In addition, a PE-10 tube (BD Biosciences) ensheathed by PE50 connected to an osmotic minipump (Model # 1002; ALZET Osmotic Pumps, Cupertino, CA, USA) was implanted at the left end (AP 1.0 mm; ML −1.5 mm; DV −3 mm). The minipump was filled with 100 μL of solution with or without CXCL12 (1 μg). The vehicle solution was PBS with 0.1 % bovine serum albumin. The residual amount in the minipump was checked on D7 to ensure the delivery. The Alzet #1002 minipump delivers 0.25 μL every hour, and the residue was approximately 50 μL on D7.

The administration approach of CXCL12 (Millipore) to the targeted region was verified using immunohistology in twelve rats. Six received CXCL12 minipump infusion, while the other received vehicle. The infusion dosage of CXCL12 was 60 ng/day. This concentration was determined empirically accordingly to our pilot study. The dosage was much lower as compared to those of the previous studies (Shyu et al. 2008; Shin et al. 2011), which used a daily dosage of up to 4 μg. A possible explanation for a lower concentration was our use of minipump for continuous infusion. Given the fact that CXCL12 has a very short half-life (25.8 ± 4.6 min) in the circulation (Rempel et al. 2000), bolus injection might be less effective than slow delivery and thus required much higher dosage.

### PB staining in NPCs

1 × 10^5^ cells were spread on the glass slide by cytocentrifugation (Model: Cytospin-4, Shandon, Thermo Scientific, Asheville, NC, USA). After air drying, cells were fixed with 4 % paraformaldehyde (Sigma-Aldrich), washed, incubated for 30 min with 10 % potassium ferrocyanide (Sigma-Aldrich) in 20 % HCl, washed again, and counterstained with nuclear fast red (Sigma-Aldrich). The slides were then dehydrated with 70, 95, and 100 % ethanol for 1 min in each, cleared in two changes of xylene for 3 min, and mounted using DPX (Sigma-Aldrich). The slides were imaged under bright field at 100× magnification, and cells exhibiting blue intracellular particles were considered positive.

### The proliferation assay in NPCs

MPIO-labeled and unlabeled NPCs were cultured in 96-well plates (BD Biosciences) at a density of 1,000 cells per well in 100 µl of the N2 medium for 7 days. Ten microliter of MTT (Roche Applied Science, Indianapolis, IN, USA) in PBS at 5 mg/mL was added to each well. After 4 h of incubation, 100 µl of a 10 % (w/v) sodium dodecyl sulfate solution (Roche Applied Science) in 0.01 N HCl was added to lyse the cells and to solubilize the formazan crystals. After 12 h of dissolution of the formazan crystals, the absorbance of the formazan product was measured using a Spectra Max 340PC384 microplate spectrophotometer (Molecular Devices, Downingtown, PA, USA) at a wavelength of 570 nm with 750 nm as the reference. The assay was repeated three times in quadruplicates.

### The differentiation assay in NPCs

1 × 10^4^ labeled and unlabeled NPCs were seeded onto 12-mm coverslips (Paul Marienfeld GmbH & Co. KG, Lauda-Königshofen, Germany) coated with 20 μg/mL of laminin (Millipore, Billerica, MA, USA). Differentiation was induced by growing the cells in 1 % FBS supplemented N2 medium devoid of EGF and FGF. After 7 days, cells were fixed with 4 % paraformaldehyde for 20 min and stained by neuronal, astrocyte, and oligodendrocyte markers. After blocking of nonspecific staining, the NPCs were incubated overnight at 4 °C with one of the marker antibodies, including rabbit anti-monoclonal GFAP (1:200 dilution, Abcam, Cambridge, MA, USA), mouse anti-Oligodendrocyte (1:200 dilution, Millipore, USA), and mouse anti-beta III tubulin (1:200 dilution, AbD Serotec, Raleigh, NC, USA). This was followed by incubation with secondary antibodies (donkey anti-rabbit or donkey anti-mouse from Jackson Immunoresearch Laboratories, West Grove, PA, USA) conjugated with rhodamine for 1 h in dark. The antibodies were diluted in PBS containing 10 % normal donkey serum (NDS) (Sigma-Aldrich) and 0.1 % Triton-X-100 (Fisher Scientific, Pittsburgh, PA, USA). The labeled cells were further stained with PB enhanced by DAB (Sigma-Aldrich). The PB-stained cells were reacted with 0.014 % diaminobenzidine (DAB) activated by 0.03 % hydrogen peroxide (Merck, Whitehouse Station, NJ, USA) for 5 min.

### In vivo MRI tracking of the implanted cells in the brain

Eleven rats engrafted with MPIO-labeled NPCs were used for MRI. Six were treated with CXCL12 while five with vehicle. The migration of the engrafted cells along the callosal pathway was tracked by 3D T2*-WI repeatedly at D0 (the engraftment day), D1, D3, and D7. Six CXCL12-treated and five vehicle-treated animals were scanned. For MRI, each rat was anesthetized by 2 % isoflurane in oxygen flowed at 1–2 L/min. MR images were acquired using a 4.7-T spectrometer (Biospec 47/40, Bruker, Karlsruhe, Germany) with a 72-mm volume coil as the RF transmitter and a quadrature surface coil placed on the head as the receiver. Rats are fixed in a prone position during scanning. A 3D gradient echo sequence was used with time of repetition = 30.0 ms, time to echo = 15.0 ms, flip angle = 30°, number of average = 4, field of view = 25.6 × 25.6 × 25.6 mm, bandwidth = 45,045 Hz, matrix = 256 × 128 × 128 zero-filled to 256 × 256 × 256, and total scan time = 32 m 46 s.

### MRI data analysis

The migration distance and volume were calculated on T2*-WI using Avizo (Visualization Sciences Group 3D, Burlington, MA, USA). The pixels showing hypointensity in contrast to the background were selected by thresholding at the lowest 25 % of the signal intensities and used for measurement. The distance or volume along the callosal pathway was defined as the distance or area from the edge of the graft proximal to the CXCL12 infusion pump to the end of the migration path toward the infusion site. The distance of the migratory path was calculated from an average of 6 slices covering the graft. The volume of the migratory path was the total summed voxel size from the defined migratory path.

### Histology and immunohistology

The animals were perfused with 4 % paraformaldehyde. The brain was sectioned at a thickness of 50 μm. For PB staining, the sections were treated with 2 % (w/v) potassium ferrocyanide solution mixed with 2 N HCl at a 1:1 ratio for 15 min and then counterstained with nuclear fast red (Sigma-Aldrich) for 3 min. For immunohistology, after being pretreated with PBS containing 0.3 % H_2_O_2_ and 0.1 % NaN3 (Sigma-Aldrich), the sections were incubated with mouse anti-CXCL12 (1:100; MAB#350, R&D systems, Minneapolis, MN, USA) or mouse anti-nestin (1:100) diluted in PBS containing 0.3 % Triton-X and 5 % normal donkey serum overnight at 4 °C, followed by incubation in biotin-conjugated donkey anti-mouse IgG (1:200; Dako, Glostrup, Denmark) for 1 h. The sections were immersed in PBS with 0.3 % Triton-X and avidin–biotin complex (1:500) (Vector Labs, Burlingame, CA, USA) at room temperature for 3 h. The sections were then stained in 0.05 M Tris buffer with 0.025 % (w/v) DAB, 1.5 % (w/v) nickel ammonium sulfate (Honeywell Riedel-de Haen, Seelze, Germany) (omitted when staining nestin) and 0.024 % H_2_O_2_ for 2–5 min until the desired dark-purple color had developed. The sections were then washed, mounted on coated slides, dehydrated, and coverslipped with DPX. The CXCL12-stained sections were photographed under a light microscope (BX51, Olympus, Japan). CXCL12 immunoreactivity was observed as particles surrounding cells or in cells per se. The quantification was done by counting the number of particles that are stained positive for CXCL12.

### Statistical analysis

The differences between CXCL12- and vehicle-treated groups were compared using *t* tests or repeated-measure ANOVAs followed by Fisher’s post hoc tests for multiple comparison. The level of statistical significance was set at *p* < 0.05.

### Data presentation

**Fig. 1:**
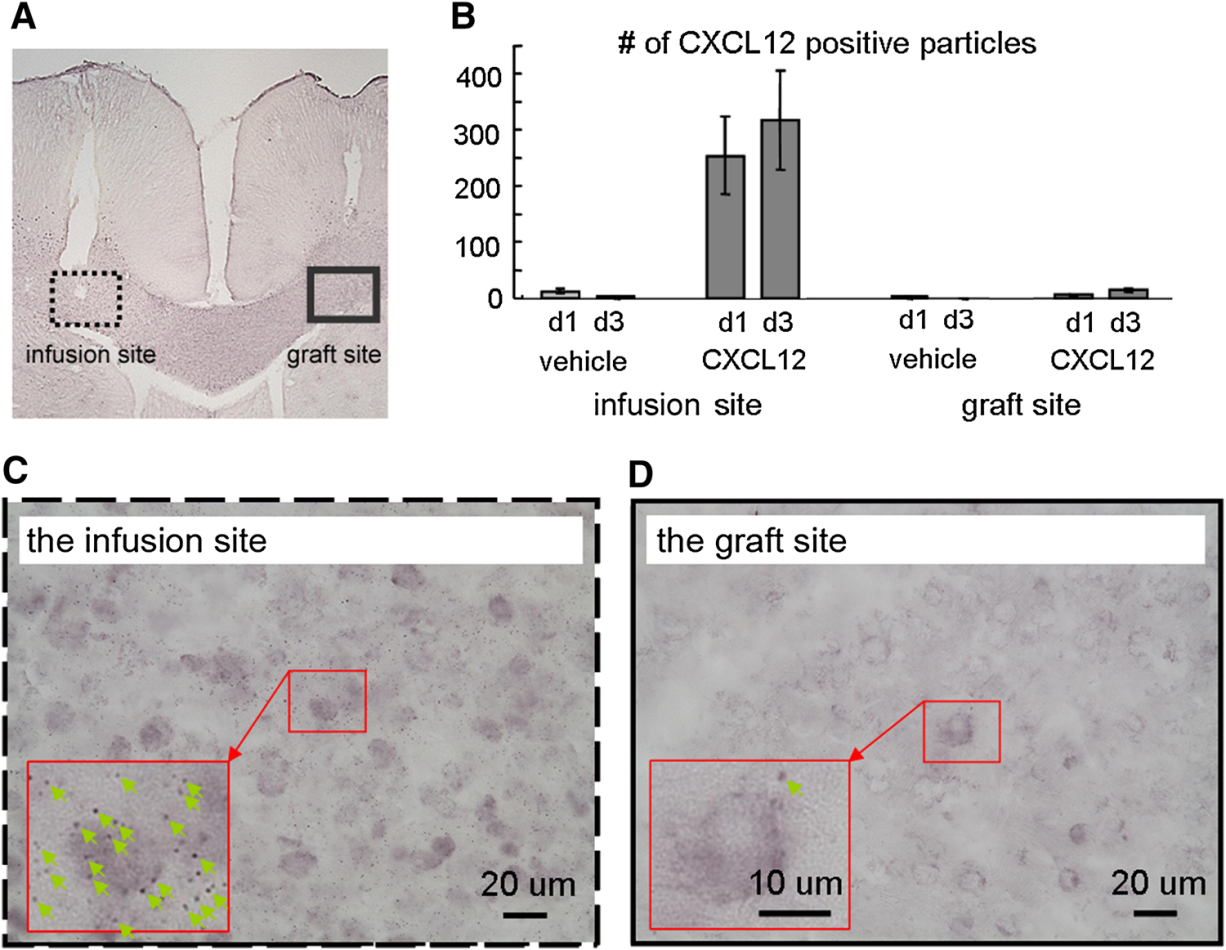
CXCL12 levels assessed in the spontaneous and treated conditions. **a** A brain slice showing the minipump tubing tract and cell graft. **b** The CXCL12-positive particles surrounding cells were counted. For the CXCL12-treated group, CXCL12 immunoreactivity was significantly elevated at the infusion site after 1 day of infusion and increased further after 3 days. **c** CXCL12 infusion caused significant immunoreactivity nearby the infusion site. **d** Little immunoreactivity was observed at the graft site. The spontaneous and treated groups contained six rats each

The image data (photomicrographs in Figs. 1a, c, 5, 6; MR images in Fig. 3) are from single representative animals from each of the groups, while the group-averaged data are shown in Figs. 1b and 4.

## Results

### CXCL12 levels assessed in the spontaneous and treated conditions

Figure 1a presents a brain slice showing the NPC graft and the CXCL12 infusion tract. Figure 1b presents the quantitative data of CXCL12 immunoreactivity expressed by the count of the CXCL12-positive particles at the infusion and graft sites between the groups across time. In the spontaneous (vehicle-treated) condition, the CXCL12 level was minimal in the CC of the adult rat. By contrast, in the CXCL12 infusion condition, CXCL12 levels were significantly higher at the infusion site after 1 day of infusion and showed an increasing trend after 3 days. Figure 1c and d shows the magnified views of the infusion site and the graft site of the CXCL12 infusion condition, respectively. More immunoreactivity was observed at the infusion site. Many cells were surrounded by particles immunoreactive for CXCL12.

### CXCR4 expression in NPCs verified by flow cytometry

**Fig. 2:**
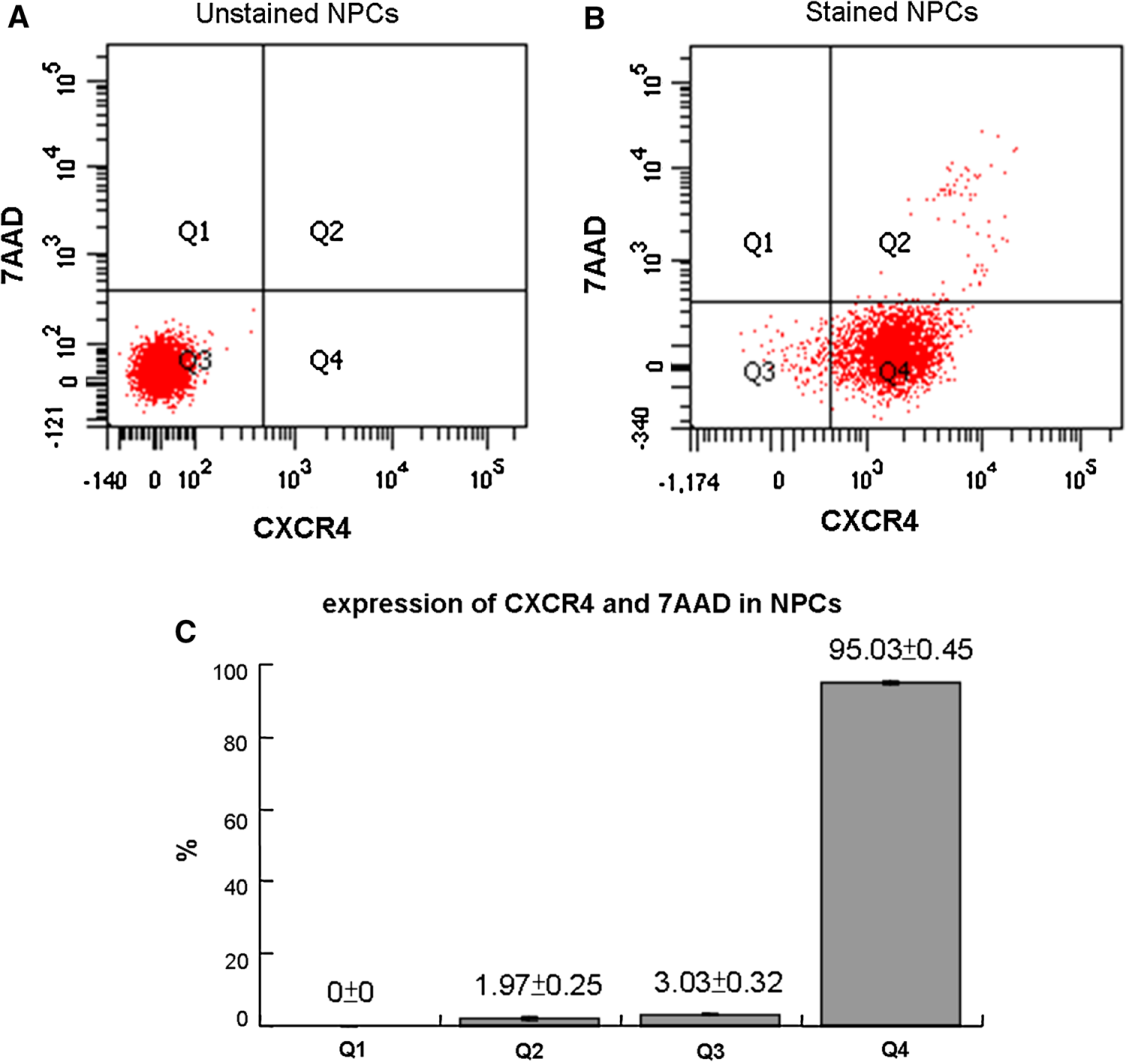
CXCR4 expression in NPCs verified by flow cytometry. **a** Gating for positive or non-fluorescence was done on unstained NPCs. The levels of the fluorescence were minimal. **b** The majority of the NPCs stained with the CXCR4 antibody and 7AAD fell within Q4 (CXCR4+/7AAD−). **c** More than 95 % of the NPCs were viable and CXCR4 positive. The values are expressed as mean ± standard deviation

The NPCs were stained by the antibody against CXCR4 and 7-AAD as the viability marker. Gating was done on unstained NPCs as shown in Fig. 2a. This generated four divisions as denoted. Figure 2b shows that the majority of the stained NPCs fell within Q4 (CXCR4+/7AAD−). The viable, CXCR4-positive population was more than 95 % as shown in Fig. 2c.

### Cellular MRI shows CXCL12 induced stronger migration of NPCs along the CC in vivo

**Fig. 3:**
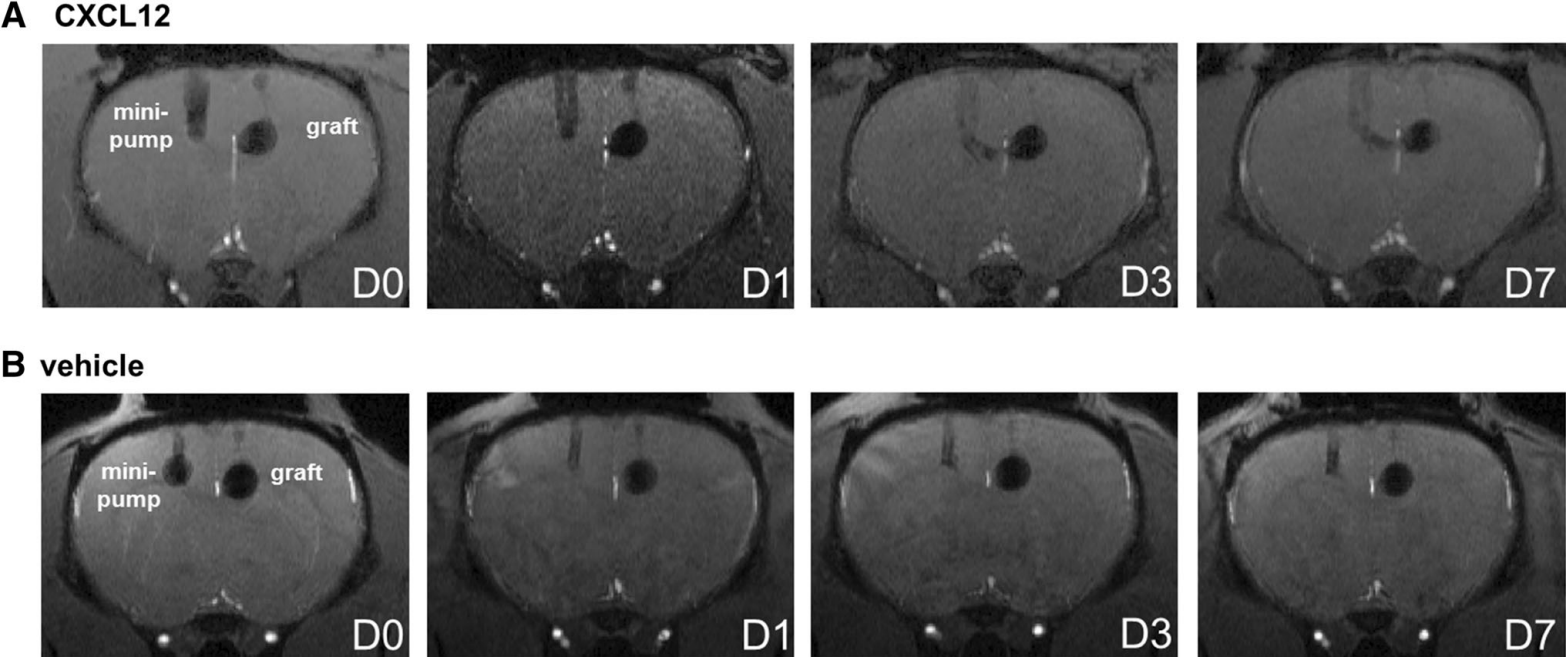
Cellular MRI shows CXCL12 induced stronger migration of NPCs along the CC in vivo. **a** The infusion tube appeared on the left while the NPC graft on the right as labeled. On D1, CXCL12 infusion led to a slight hypointense stream directed toward the side of the infusion tube. On D3, the migratory path extended from the NPC graft toward the infusion site. On D7, the path became more visible due to increased hypointensities. **b** Infusion of vehicle induced no obvious signal change along the callosal pathway with time. The vehicle- and CXCL12-treated groups contained five and six rats, respectively

Axial 3D T2*-WI images were acquired on D0, D1, D3, and D7 after the transplantation and infusion surgery. Figure 3a shows the representative images at the four different time points merged from four slices that covered the CXCL12 infusion site and the migration path. The infusion minipump tubing appeared on the left while the NPC graft on the right as labeled. On D1, a stream-like hypointensity directed toward the side of the infusion tube was observed. On D3, the migratory path extended further from the NPC graft site. On D7, the path became more obvious due to increased hypointensities. In contrast, no obvious signal changes along the callosal pathway across time was seen in the spontaneous condition (i.e., the vehicle group), as seen in Fig. 3b. To rule out the possibility that the group difference was caused by a positional bias in the graft placement, the distance between the center of the graft and the interhemispherical midline was measured for each graft, and compared between the vehicle- and CXCL12-treated groups; no significant difference was detected (mean ± SD: 1.11 ± 0.24 vs. 1.19 ± 0.15 mm, respectively).

### Measuring spontaneous and induced migration along the CC on cellular MRI

**Fig. 4:**
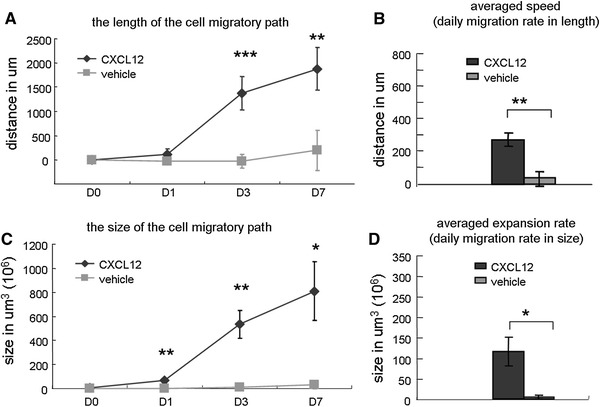
Measuring spontaneous and induced migration along the CC on cellular MRI. **a** In length, CXCL12 induced the NPCs to migrate with time over an extensive range from the grafted area. CXCL12-induced migration was nearly nine times as long as the vehicle-induced migration. **b** The averaged migration speeds were 269 ± 41 and 29 ± 45 μm/day for the CXCL12 and vehicle groups, respectively. **c** The migratory path increased in size with time. The size of CXCL12-induced migration was 28.7 times as large as vehicle-induced migration (i.e., spontaneous). **d** The average expansion rates from D0 to D7 were 115 ± 35 × 106 and 4 ± 4 × 106 μm^3^/day for the CXCL12 and vehicle groups, respectively

The migration of NPCs induced by CXCL12 was characterized by several parameters. In *length*, CXCL12 induced the NPCs to move over an extensive range from the grafted area with time. As depicted in Fig. 4a, on D7 after NPC engraftment, the average migration was up to 1,881 μm. In contrast, the spontaneous migration observed in the vehicle-treated group was mere 200 μm. CXCL12-induced migration was nearly nine times as long as vehicle-induced migration. The statistical differences were detected by repeated-measures ANOVA followed by Fisher’s post hoc tests [*F*(1, 9) = 36.82, *p* < 0.001; D3, *p* < 0.0001; D7, *p* < 0.01]. The averaged *migration speeds*, calculated from the migration distance over time, were 269 ± 41 and 29 ± 45 μm/day for the CXCL12 and vehicle groups, respectively (*p* < 0.01) (Fig. 4b).

The migratory path was found to increase in *size* with time as shown in Fig. 4c. The size of CXCL12-induced migration was 28.7 times as large as that of the vehicle-induced (i.e., spontaneous). The statistical differences were detected by repeated-measures ANOVA followed by Fisher’s post hoc tests [*F*(1, 9) = 12.57, *p* < 0.01; D1, *p* < 0.01; D3, *p* < 0.01; D7, *p* < 0.05]. The expansion rates, calculated from the size increments over time, are shown in Fig. 4d. The averaged *expansion rates* from D0 to D7 were 115 ± 35 × 10^6^ and 4 ± 4 × 10^6^ μm^3^/day for the CXCL12 and vehicle groups, respectively (*p* < 0.05).

### Corroboration of the chemotaxis by PB staining

**Fig. 5:**
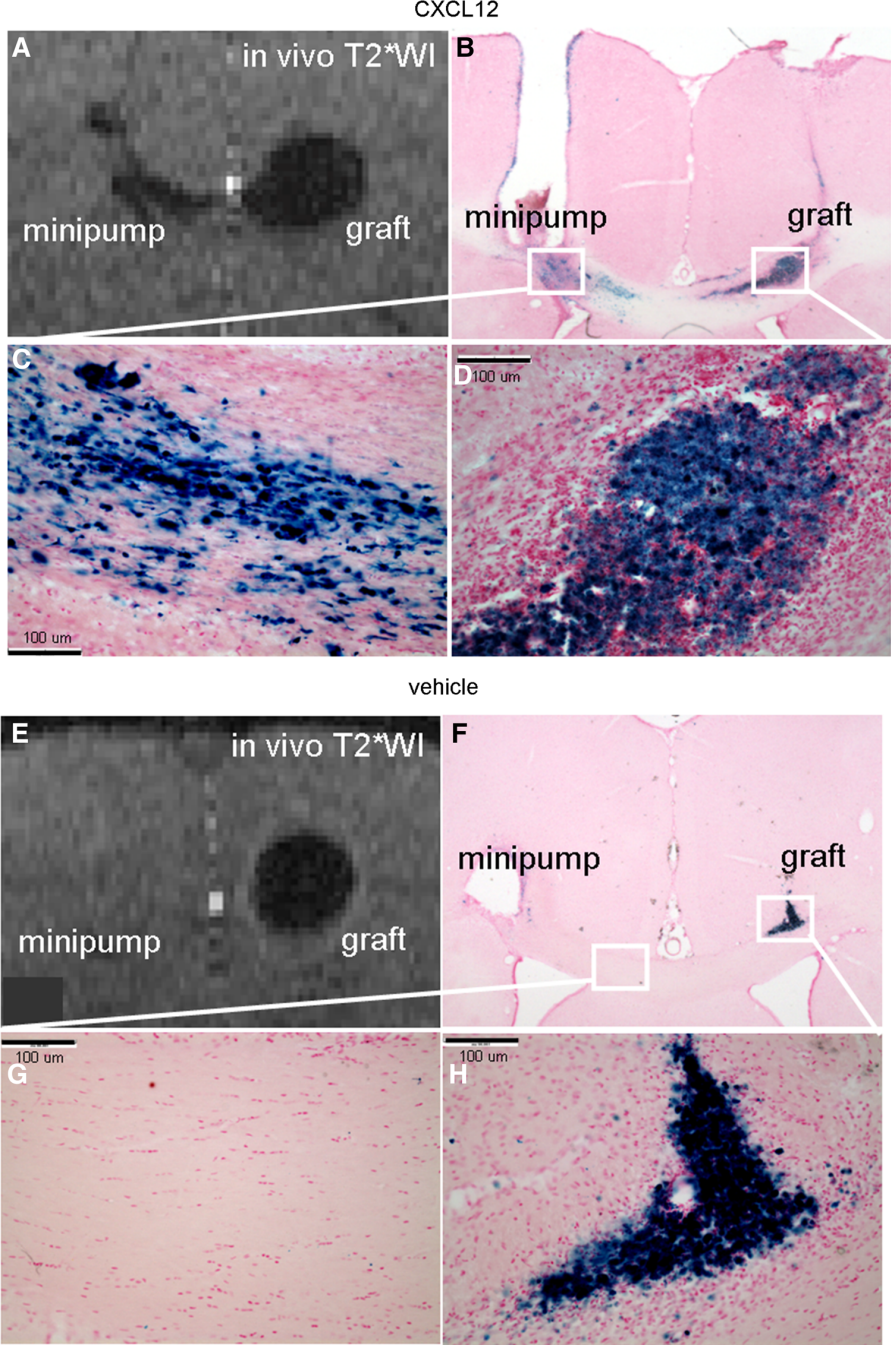
Corroboration of the chemotaxis by PB staining. **a** The NPC migration induced by CXCL12 infusion seen as the hypointensities on T2*WI. **b** The PB staining concurred with the hypointensities on T2*WI. **c** A magnified view of the migratory path toward the target. **d** A view of the graft. **e** T2*WI of the group with vehicle infusion showed little migration. **f** PB-stained cells were mainly distributed in the graft site. **g** A magnified view of the corpus callosum. **h** A view of the graft

PB staining was used to confirm the presence of MPIO-labeled NPCs in the brain sections. NPC migration induced by CXCL12 infusion is visible as hypointensities on T2*-WI (Fig. 5a). Figure 5b depicts the distribution of PB-stained cells corresponding to the graft, migratory path, and the minipump. The PB staining matched with the hypointensities on T2*-WI. Magnified views of the migratory path toward the target and of the graft are shown in Fig. 5c and d, respectively. In the case of vehicle infusion, minimal migration was observed (Fig. 5e). PB-stained cells were mainly observed in the graft site as shown in Fig. 5f. Magnified views of the corpus callosum and the graft are shown in Fig. 5g and h, respectively.

### Corroboration of the progenitor identity by nestin immunohistology

**Fig. 6:**
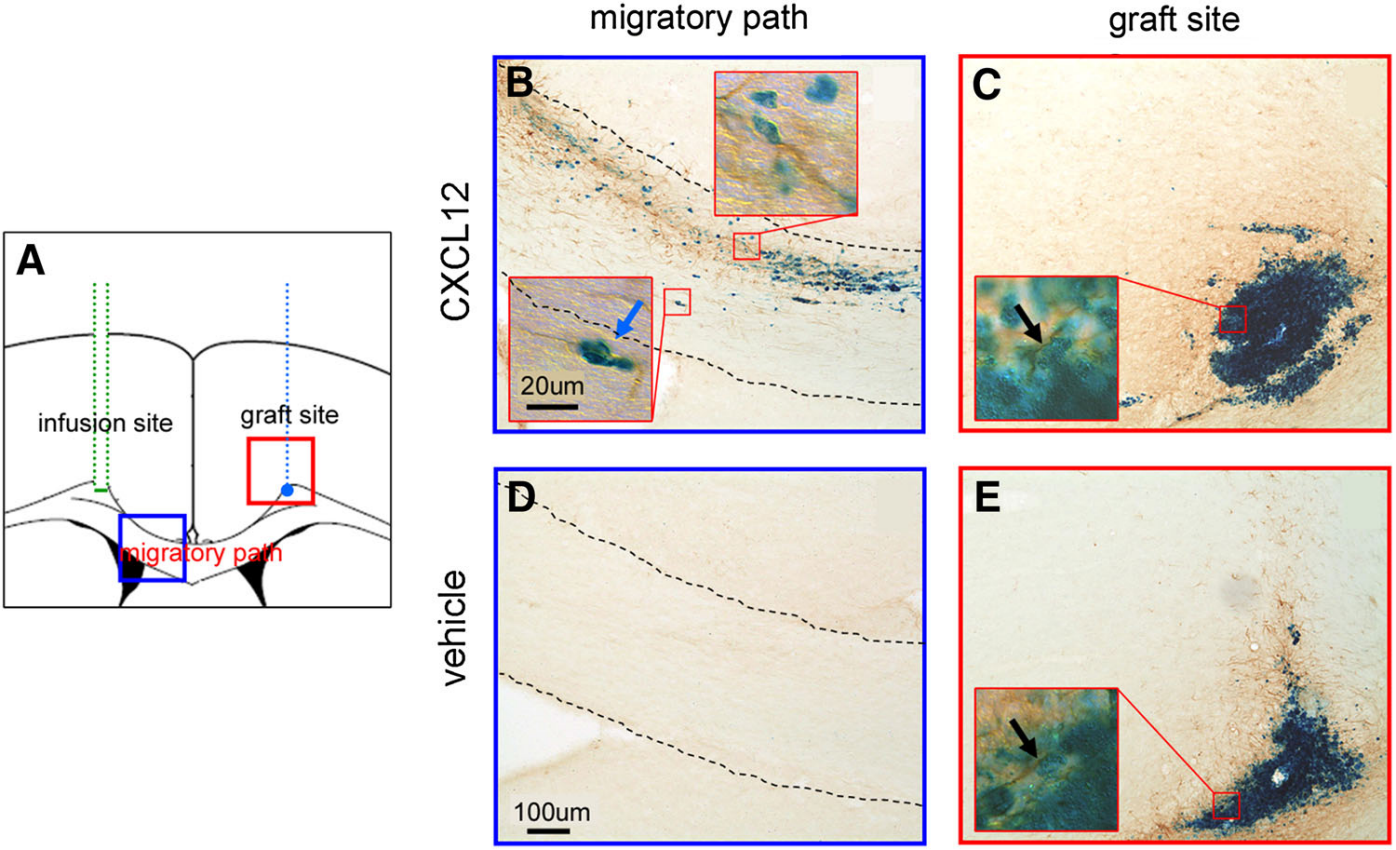
Corroboration of the progenitor identity by nestin immunohistology. **a** The diagram of the infusion tube, migratory path, and graft. The areas nearby the graft and the migratory path marked by *squares* were photomicrographed. **b** A closer view of the migrating NPCs taken from the CXCL12-treated group. The NPCs were observed with both PB and nestin immunoreactivity. **c** The NPCs of the graft treated with CXCL12 infusion were positive for both nestin and PB. **d** Little migration occurred in the vehicle-treated group. **e** The graft view from the vehicle-treated group. Neither PB staining nor nestin immunoreactivity was observed beyond the graft site

Figure 6a shows the diagram of the infusion minipump, migratory path, and graft. The areas nearby the migratory path and graft marked by squares were photomicrographed. Figure 6b shows a closer view of the migrating NPCs. The NPCs were observed with both PB and nestin immunoreactivity. Figure 6c shows the graft treated with CXCL12 infusion. NPCs were positive for both nestin and PB. An additional photomicrograph at the off-center position of the graft is shown in Supplemental Fig S1, which reveals more distinct nestin immunoreactivity. In the vehicle-treated group, little migration occurred as shown in Fig. 6d. Figure 6e shows the graft treated with vehicle infusion. Positive staining was mainly seen at the graft site. Thus, neither PB staining nor nestin immunoreactivity was observed beyond the graft site. Regarding in vivo differentiation, it seems that the CXCL12 treatment regulated the fate of the transplanted NPCs, as shown in Supplemental Fig S2.

### Non-adverse effects on NPCs caused by magnetic labeling

**Fig. 7:**
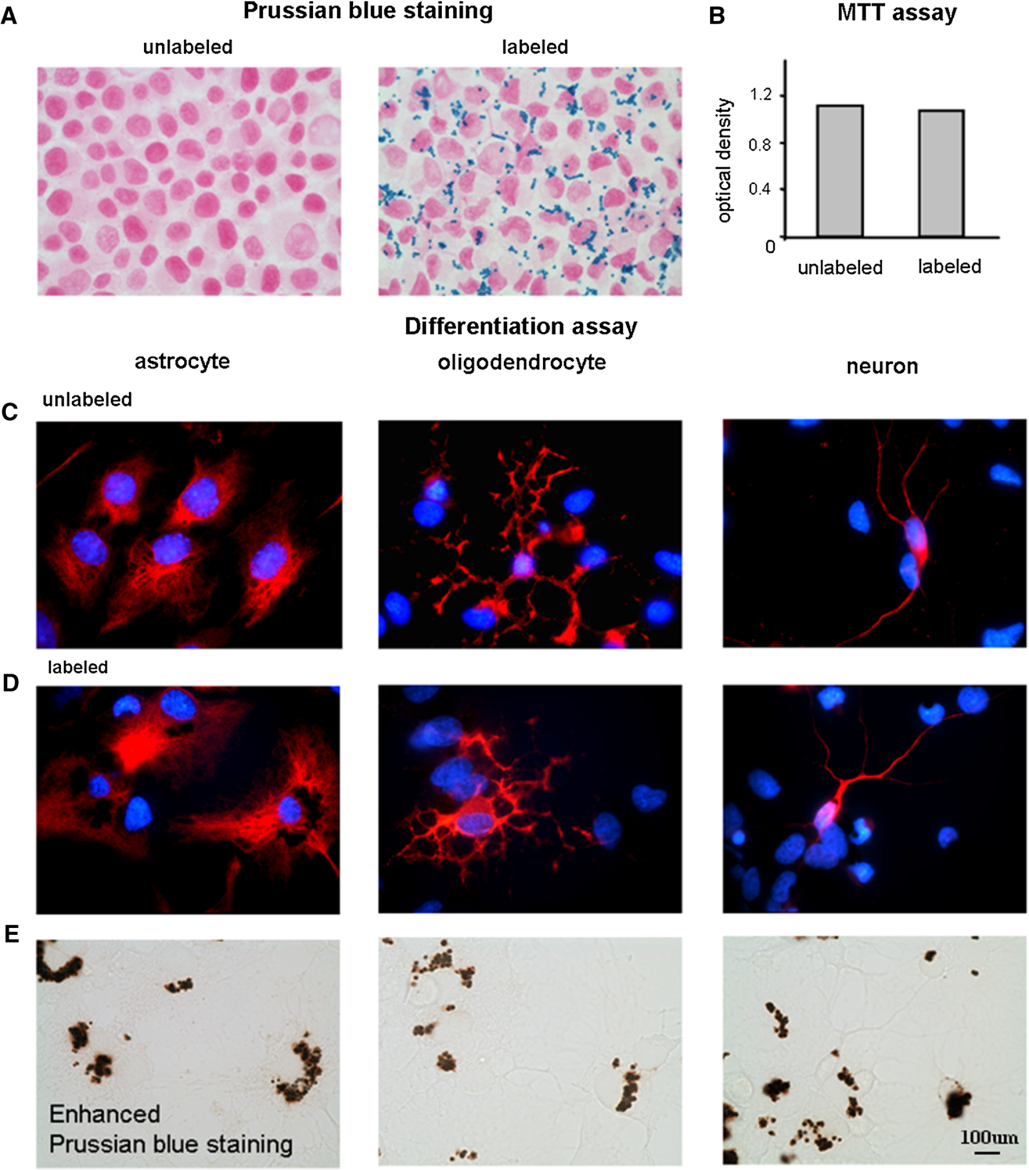
NPCs labeled with MPIO retained the ability to proliferate and differentiate. **a** No positive PB staining was observed in the unlabeled NPCs while numerous PB-stained particles were found in the cytoplasm of the MPIO-labeled NPCs. **b** MTT assays indicated a similar proliferation capacity between the labeled and unlabeled NPCs. **c** Differentiation of the unlabeled NPCs into astrocytes, oligodendrocytes, and neurons. **d** The labeled NPCs preserved the differentiation capacity. **e** The iron content in the labeled NPCs was stained by diaminobenzidine-enhanced PB. The results indicated that NPCs labeled with MPIO retained the ability to proliferate and differentiate

As shown in Fig. 7a, numerous PB-stained particles were found in the cytoplasm in the labeled NPCs. In Fig. 7b, MTT assays indicated no significant difference in cell proliferation between the unlabeled and labeled NPCs. Differentiation was also examined. Both the unlabeled and labeled NPCs exhibited similar capacities for cellular differentiation as shown in Fig. 7c and d, respectively. The iron content of the labeled NPCs was stained by DAB-enhanced PB as shown in Fig. 7e. Note that MPIO labeling tended to obstruct the fluorescence shown in Fig. 7d, leading to a misimpression of weaker immunoreactivity in the labeled NPCs. The results indicate that NPCs labeled with MPIO retained the ability to proliferate and differentiate.

## Discussion

The present study investigated whether neurochemical attraction via CXCL12/CXCR4 signaling is responsible for the efficient migration along the CC. It was found that, in the spontaneous (vehicle) condition, little CXCL12 immunoreactivity was detected in the CC, and this corresponded to the minimal migration seen during the monitoring period. The results indicate that NPCs had minimal mobility along the white matter tracts when the CXCL12 concentration was not up-regulated. By contrast, continuous CXCL12 infusion led to elevated CXCL12 levels in the CC, and this significantly increased migration of the transplanted NPCs through the CC. Continuous CXCL12 administration at 60 ng/day resulted in an averaged migration speed at 269 ± 41 μm/day or an averaged expansion rate of 115 ± 35 × 10^6^ μm^3^/day into the CC. CXCL12 augmented the average migration distance by a factor of up to 9, and the overall volume by a factor of up to 29 compared to spontaneous migration. This indicates that CXCL12/CXCR4 signaling is a key mechanism whereby the white matter tracts are utilized by NPCs for efficient movement. It is possible that high chemokine attraction in a structure with high directionality is an important combinational factor for efficient NPC migration across a long distance within the brain.

NPC migration into the white matter structures is well known in brain disorders involving axonal injuries or demyelination (Nam et al. 2007; Zhang et al. 2003; Hoehn et al. 2002; Banisadr et al. 2011). In ischemic stroke, where white matter lesion is sometimes reported, engrafted NPCs migrated all the way across the callosal pathway to the ischemic lesioned area in a rat stroke model (Hoehn et al. 2002). The mean migration speed was estimated to range from 360 μm/day (Kim et al. 2004) to 50–80 μm/h (Zhang et al. 2003). In consistence with the findings, the expression of CXCL12 within the white matter structures was found to increase following hypoxic ischemic injury (Miller et al. 2005). In experimental autoimmune encephalomyelitis, NPCs engrafted in the lateral ventricle migrated up to 500 μm through the white matter tracts (Ben-Hur et al. 2003). The migration speed of endogenous NPCs is approximately 50–80 μm/h in multiple sclerosis (Nam et al. 2007). Evidence has shown that the NPC migration through white matter in experimental autoimmune encephalomyelitis was also based upon the CXCL12/CXCR4 signaling (Banisadr et al. 2011).

Nevertheless, NPCs migration into the white matter structures is not a scenario limited to white matter dysfunctions. That is, such migration is not necessarily intended for regional tissue repair. The present study and several previous reports indicate that migration into white matter also occurs in healthy subjects during the neonatal stage and in the adulthood. In these cases, the white matter structures may mainly serve as a migratory road for the cells. In neonatal rats, MRI revealed that endogenous NPCs labeled by MPIO migrated through white matter toward the cortex (Yang et al. 2009). In some cases, white matter in the vicinity of the subventricular zone is the only structure where endogenous migrating NPCs from the SVZ can be found (Suzuki and Goldman 2003). In the adult rat brain, there is a small degree of spontaneous migration along the white matter tracts. It was previously reported that NPCs engrafted in the corpus callosum migrated at the speed of 50–70 μm/day (Flexman et al. 2011). The present study reported the spontaneous migration speed along the callosal pathway being 29 μm/day over the 7-day tracking period, lower than that reported earlier (Flexman et al. 2011). This difference may arise from variations in the experimental protocols, including the cell types, the labeling particles used, and the imaging resolution.

It needs to be addressed whether the dosage of CXCL12 at 60 ng/day used in the present study is a physiologically relevant concentration. Previous in vitro studies indicated that CXCL12 concentrations at 100 to 1,000 ng/mL showed approximately 1.6 to 1.75-folds of increases in migration in the Boyden chamber assay (Imitola et al. 2004). This concentration range was empirically tested in our pilot study. It was found that, to induce in vivo migration in the brain, equivalent dosages via continuous infusion are required. Lower concentrations produced little migratory effects as found in our pilot study. But the administered dose range was in a sharp contrast to the reported secreted levels of endogenous CXCL12 in brain injuries, which falls within the ranges of tens to hundreds of picogram per milliliter or milligram. The disparity may arise from the difference in the measurement. CXCL12 has a very short half-life (25.8 ± 4.6 min in the circulation) (Rempel et al. 2000). Since CXCL12 secretion was measured at a fixed time point as the concentration to the tissue volume/mass, the level could be significantly underestimated when extrapolated to daily estimation. In addition, the short half-life may also affect the sensitivity and detectability of the measuring method, enzyme-linked immuno-sorbent assay (ELISA). If the factors of time and sensitivity loss are both considered, the dosage given in the present study could be within a physiologically reasonable range close to the secreted levels.

CXCL12 is also known for its influences on cell survival. To understand whether our approach produced effects on the survival of the engrafted NPCs, additional TUNEL staining was performed. But no significant difference in the percentage of TUNEL stained cells was observed between the two groups (31.8 ± 17.4 % in CXCL12 vs. 28 ± 10.2 % in vehicle). The findings further indicate that the effects of CXCL12 on the engrafted NPCs were mainly on the migratory behavior.

One major limitation associated with the use of cellular MRI tracking is that gradual loss of iron oxide nanoparticles, which leads to lowered detectability (Berman et al. 2011). Cell division or spontaneous exocytosis is responsible for such labeling loss. In the present study, it was found that use of large-sized labeling particles alleviates this problem. Despite the criticisms regarding the use of iron particles in cell tracking, this approach was not found to be disappointing. The use of MPIO nanoparticles appeared to rectify some of the problems reported with the use of smaller nanoparticles such as superparamagnetic iron oxide (Shapiro et al. 2005; Neri et al. 2008). A very high spatial correspondence between nestin immunoreactivity and PB staining was observed. At the NPC graft site, the engrafted cells were heavily double stained with nestin and PB. The distribution of the staining matched well with the appearance on MRI. This indicates that most of the engrafted NPCs retained the MPIO labeling even after migrating away from the injection site. Some NPCs, observed without PB staining toward the tube site and thus not shown on MRI, could be either cells that lost labeling following engraftment, or endogenous NPCs originating from the subventricular zone or the subgranular zone. However, the occurrence of either case did not invalidate the utility of cellular MRI in tracking cells, since the correspondence between MRI and histological findings remained reasonably strong in most of the brain areas (Hoehn et al. 2002).

Related to the issue of loss of labeling, the second limitation of the cellular MRI tracking system is the MR signals supposedly arising from the labeled NPCs may be nonspecific. Upon the loss of labeling, the nanoparticles are either cleared from the region by scavenger cells or are taken up by surrounding cells. If this occurs, the hypointense signals appearing on the MRI may then represent behavior of different cells instead of those intended for observation (Berman et al. 2011). This could undermine the utility of MRI in cell tracking. However, the high correspondence among the hypointensities, PB staining, and most of the nestin staining indicates that this issue is of less concern in the present study. Very few PB-stained cells were nestin negative. Hence, at both the macro- and microscopic levels, cellular MRI appears to be a relatively reliable tool to monitor the migration of engrafted cells (Politi 2007; Lee et al. 2004; Mligiliche et al. 2005; Zhang et al. 2003, 2004).

## Conclusions

The present study aimed to understand why the white matter tracts are an efficient route for NPC migration. Cellular MRI supports the importance of CXCL12/CXCR4 signaling in facilitating NPCs migrating along the CC. In addition to the mechanistic implications, the administration of CXCL12 into the white matter structures may be developed as a strategy to promote the migration of the transplanted NPC in the future.

## Electronic supplementary material

Below is the link to the electronic supplementary material.

Supplementary material 1 (DOCX 3889 kb)

## Abbreviations

NPCs: Neural progenitor cells
CC: Corpus callosum
MPIO: Micron-sized paramagnetic iron oxide
3D: Three dimensional
T2*WI: T2*weighted imaging
PB: Prussian blue
APC: Allophycocyanin
7-AAD: 7-Aminoactinomycin-D
NDS: Normal donkey serum
DAB: Diaminobenzidine

## References

[CR1] Aboody KS, Brown A, Rainov NG, Bower KA, Liu S, Yang W, Small JE, Herrlinger U, Ourednik V, Black PM, Breakefield XO, Snyder EY (2000). Neural stem cells display extensive tropism for pathology in adult brain: evidence from intracranial gliomas. Proc Natl Acad Sci USA.

[CR2] Assaf Y, Pasternak O (2008). Diffusion tensor imaging (DTI)-based white matter mapping in brain research: a review. J Mol Neurosci.

[CR3] Banisadr G, Frederick TJ, Freitag C, Ren D, Jung H, Miller SD, Miller RJ (2011). The role of CXCR4 signaling in the migration of transplanted oligodendrocyte progenitors into the cerebral white matter. Neurobiol Dis.

[CR4] Belmadani A, Tran PB, Ren D, Assimacopoulos S, Grove EA, Miller RJ (2005). The chemokine stromal cell-derived factor-1 regulates the migration of sensory neuron progenitors. J Neurosci.

[CR5] Belmadani A, Tran PB, Ren D, Miller RJ (2006). Chemokines regulate the migration of neural progenitors to sites of neuroinflammation. J Neurosci.

[CR6] Ben-Hur T, Einstein O, Mizrachi-Kol R, Ben-Menachem O, Reinhartz E, Karussis D, Abramsky O (2003). Transplanted multipotential neural precursor cells migrate into the inflamed white matter in response to experimental autoimmune encephalomyelitis. Glia.

[CR7] Berman SC, Galpoththawela C, Gilad AA, Bulte JW, Walczak P (2011). Long-term MR cell tracking of neural stem cells grafted in immunocompetent versus immunodeficient mice reveals distinct differences in contrast between live and dead cells. Magn Reson Med.

[CR8] Bhattacharyya BJ, Banisadr G, Jung H, Ren D, Cronshaw DG, Zou Y, Miller RJ (2008). The chemokine stromal cell-derived factor-1 regulates GABAergic inputs to neural progenitors in the postnatal dentate gyrus. J Neurosci.

[CR9] Flexman JA, Cross DJ, Tran LN, Sasaki T, Kim Y, Minoshima S (2011) Quantitative analysis of neural stem cell migration and tracer clearance in the rat brain by MRI. Mol Imaging Biol 13(1):104–111 10.1007/s11307-010-0311-3 20440567

[CR10] Hoehn M, Kustermann E, Blunk J, Wiedermann D, Trapp T, Wecker S, Focking M, Arnold H, Hescheler J, Fleischmann BK, Schwindt W, Buhrle C (2002). Monitoring of implanted stem cell migration in vivo: a highly resolved in vivo magnetic resonance imaging investigation of experimental stroke in rat. Proc Natl Acad Sci USA.

[CR11] Imitola J, Raddassi K, Park KI, Mueller FJ, Nieto M, Teng YD, Frenkel D, Li J, Sidman RL, Walsh CA, Snyder EY, Khoury SJ (2004). Directed migration of neural stem cells to sites of CNS injury by the stromal cell-derived factor 1alpha/CXC chemokine receptor 4 pathway. Proc Natl Acad Sci USA.

[CR12] Itoh T, Satou T, Ishida H, Nishida S, Tsubaki M, Hashimoto S, Ito H (2009). The relationship between SDF-1alpha/CXCR4 and neural stem cells appearing in damaged area after traumatic brain injury in rats. Neurol Res.

[CR13] Kim DE, Schellingerhout D, Ishii K, Shah K, Weissleder R (2004). Imaging of stem cell recruitment to ischemic infarcts in a murine model. Stroke.

[CR14] Lee IH, Bulte JW, Schweinhardt P, Douglas T, Trifunovski A, Hofstetter C, Olson L, Spenger C (2004). In vivo magnetic resonance tracking of olfactory ensheathing glia grafted into the rat spinal cord. Exp Neurol.

[CR15] Liapi A, Pritchett J, Jones O, Fujii N, Parnavelas JG, Nadarajah B (2008). Stromal-derived factor 1 signalling regulates radial and tangential migration in the developing cerebral cortex. Dev Neurosci.

[CR16] Miller JT, Bartley JH, Wimborne HJ, Walker AL, Hess DC, Hill WD, Carroll JE (2005). The neuroblast and angioblast chemotaxic factor SDF-1 (CXCL12) expression is briefly up regulated by reactive astrocytes in brain following neonatal hypoxic-ischemic injury. BMC Neurosci.

[CR17] Mligiliche NL, Xu Y, Matsumoto N, Idel C (2005). Survival of neural progenitor cells from the subventricular zone of the adult rat after transplantation into the host spinal cord of the same strain of adult rat. Anat Sci Int.

[CR18] Nam SC, Kim Y, Dryanovski D, Walker A, Goings G, Woolfrey K, Kang SS, Chu C, Chenn A, Erdelyi F, Szabo G, Hockberger P, Szele FG (2007). Dynamic features of postnatal subventricular zone cell motility: a two-photon time-lapse study. J Comp Neurol.

[CR19] Neri M, Maderna C, Cavazzin C, Deidda-Vigoriti V, Politi LS, Scotti G, Marzola P, Sbarbati A, Vescovi AL, Gritti A (2008). Efficient in vitro labeling of human neural precursor cells with superparamagnetic iron oxide particles: relevance for in vivo cell tracking. Stem Cells (Dayton, Ohio).

[CR20] Peng H, Kolb R, Kennedy JE, Zheng J (2007). Differential expression of CXCL12 and CXCR4 during human fetal neural progenitor cell differentiation. J Neuroimmune Pharmacol.

[CR21] Politi LS (2007). MR-based imaging of neural stem cells. Neuroradiology.

[CR22] Rempel SA, Dudas S, Ge S, Gutierrez JA (2000). Identification and localization of the cytokine SDF1 and its receptor, CXC chemokine receptor 4, to regions of necrosis and angiogenesis in human glioblastoma. Clin Cancer Res.

[CR23] Robin AM, Zhang ZG, Wang L, Zhang RL, Katakowski M, Zhang L, Wang Y, Zhang C, Chopp M (2006). Stromal cell-derived factor 1alpha mediates neural progenitor cell motility after focal cerebral ischemia. J Cereb Blood Flow Metab.

[CR24] Shapiro EM, Skrtic S, Koretsky AP (2005). Sizing it up: cellular MRI using micron-sized iron oxide particles. Magn Reson Med.

[CR25] Shin JW, Lee JK, Lee JE, Min WK, Schuchman EH, Jin HK, Bae JS (2011). Combined effects of hematopoietic progenitor cell mobilization from bone marrow by granulocyte colony stimulating factor and AMD3100 and chemotaxis into the brain using stromal cell-derived factor-1alpha in an Alzheimer’s disease mouse model. Stem Cells (Dayton, Ohio).

[CR26] Shyu WC, Lin SZ, Yen PS, Su CY, Chen DC, Wang HJ, Li H (2008). Stromal cell-derived factor-1 alpha promotes neuroprotection, angiogenesis, and mobilization/homing of bone marrow-derived cells in stroke rats. J Pharmacol Exp Ther.

[CR27] Stumm R, Hollt V (2007). CXC chemokine receptor 4 regulates neuronal migration and axonal pathfinding in the developing nervous system: implications for neuronal regeneration in the adult brain. J Mol Endocrinol.

[CR28] Suzuki SO, Goldman JE (2003). Multiple cell populations in the early postnatal subventricular zone take distinct migratory pathways: a dynamic study of glial and neuronal progenitor migration. J Neurosci.

[CR29] Takeuchi H, Natsume A, Wakabayashi T, Aoshima C, Shimato S, Ito M, Ishii J, Maeda Y, Hara M, Kim SU, Yoshida J (2007). Intravenously transplanted human neural stem cells migrate to the injured spinal cord in adult mice in an SDF-1- and HGF-dependent manner. Neurosci Lett.

[CR30] Tiveron MC, Rossel M, Moepps B, Zhang YL, Seidenfaden R, Favor J, Konig N, Cremer H (2006). Molecular interaction between projection neuron precursors and invading interneurons via stromal-derived factor 1 (CXCL12)/CXCR4 signaling in the cortical subventricular zone/intermediate zone. J Neurosci.

[CR31] Yang J, Liu J, Niu G, Chan KC, Wang R, Liu Y, Wu EX (2009). In vivo MRI of endogenous stem/progenitor cell migration from subventricular zone in normal and injured developing brains. NeuroImage.

[CR32] Zhang SC, Wernig M, Duncan ID, Brustle O, Thomson JA (2001). In vitro differentiation of transplantable neural precursors from human embryonic stem cells. Nat Biotechnol.

[CR33] Zhang ZG, Jiang Q, Zhang R, Zhang L, Wang L, Zhang L, Arniego P, Ho KL, Chopp M (2003). Magnetic resonance imaging and neurosphere therapy of stroke in rat. Ann Neurol.

[CR34] Zhang Z, Jiang Q, Jiang F, Ding G, Zhang R, Wang L, Zhang L, Robin AM, Katakowski M, Chopp M (2004). In vivo magnetic resonance imaging tracks adult neural progenitor cell targeting of brain tumor. NeuroImage.

