# Supplementary material for: White matter tracts for the trafficking of neural progenitor cells characterized by cellular MRI and immunohistology: the role of CXCL12/CXCR4 signaling
Source: Brain Struct Funct. 2014 Apr 26;220(4):2073–85. doi: 10.1007/s00429-014-0770-4 (PMC4481304; doi:10.1007/s00429-014-0770-4)
Supplement: Supplementary file 1 — Supplementary material 1 (DOCX 3889 kb) [file 429_2014_770_MOESM1_ESM.docx]

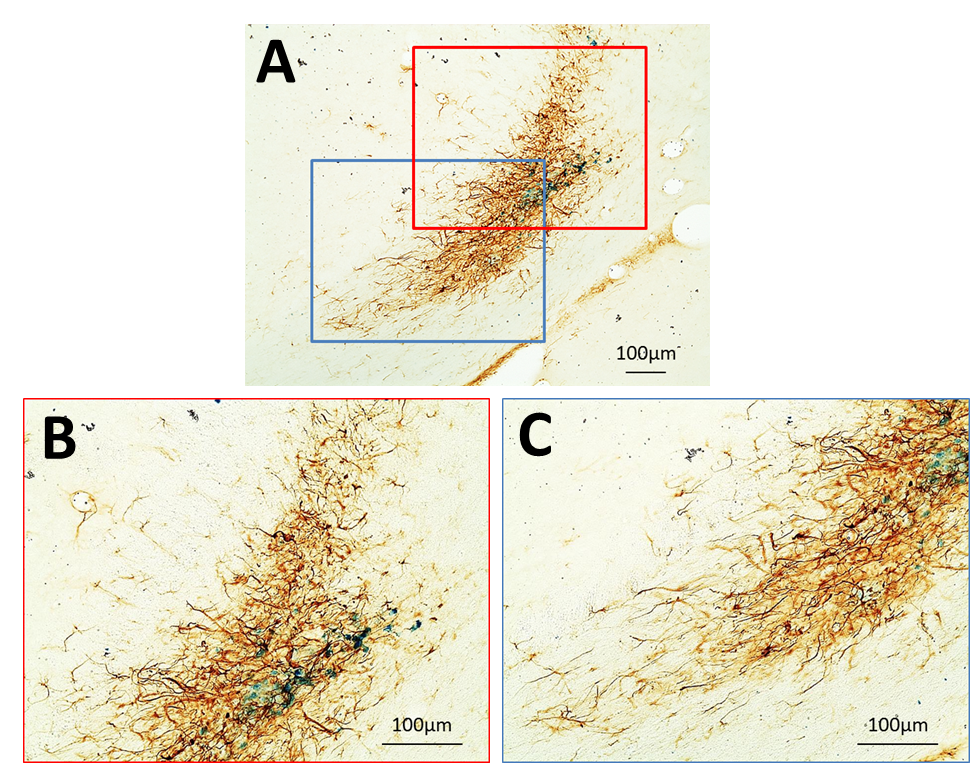


Supplemental FigS1. Nestin labeling tended to be obstructed by PB staining when both were colocalized. As shown in A, nestin labeling was more distinct within the arborous processes of the progenitor cells, while PB staining was absent in these cellular loci. B and C are enlarged views from A.


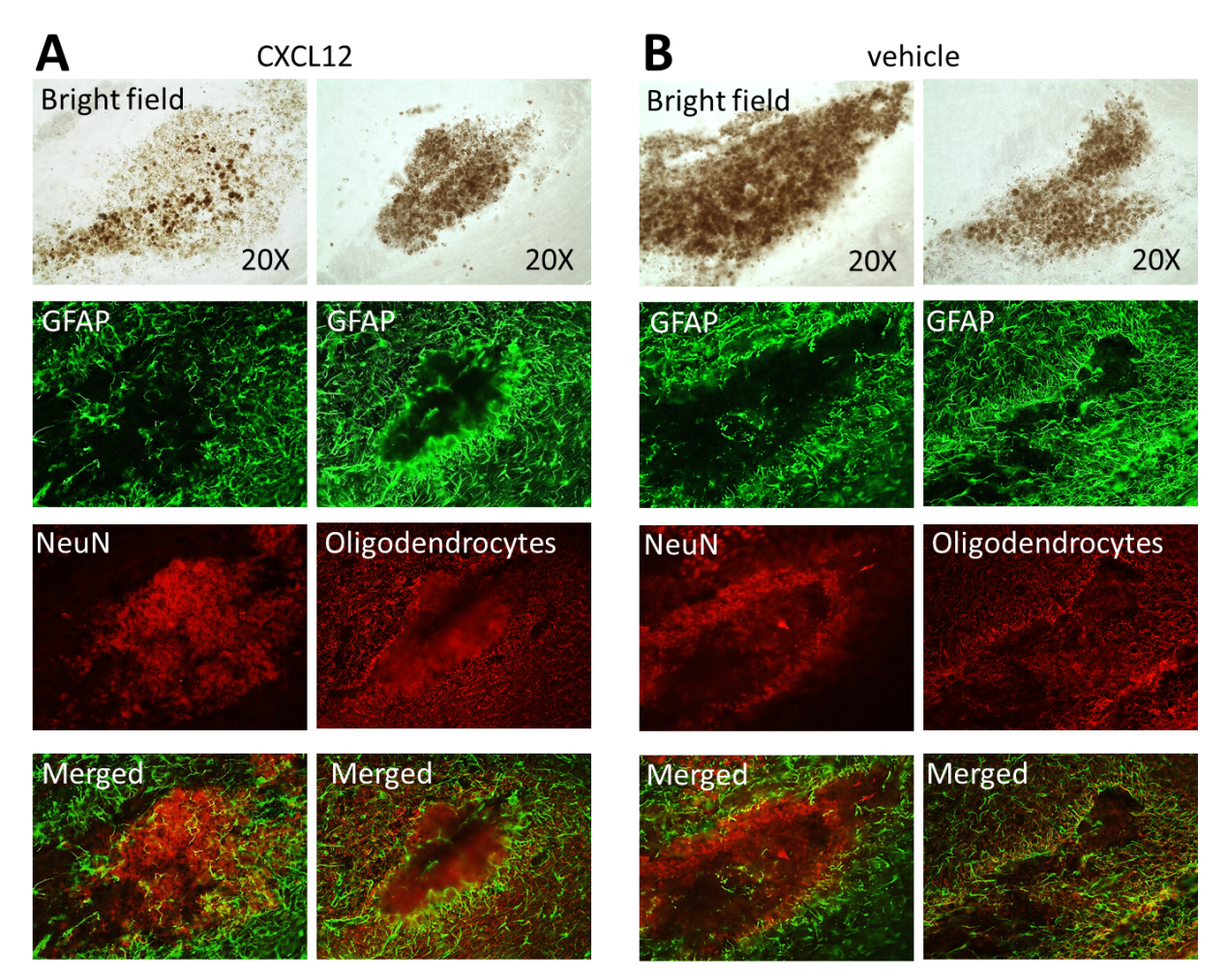


Supplemental FigS2. Differentiation patterns of the transplanted NPCs. Double-labeling of (1) GFAP and NeuN or (2) GFAP and oligodendrocytes was performed. The cell graft is seen in the bright-field views. CXCL12 treatment caused most NPCs to differentiate into neurons (strong immunofluorescence with cell morphologies), but few astrocytes (lacking immunofluorescence in the graft) or oligodendrocytes (only diffuse immunofluorescence lacking cellular morphology in the graft). In contrast, the degree of differentiation was lower in the vehicle-treated group than in the CXCL12-treated group, although the differentiated portion also mostly comprised neurons.
